# Cigarette Butt Decomposition and Associated Chemical Changes Assessed by ^13^C CPMAS NMR

**DOI:** 10.1371/journal.pone.0117393

**Published:** 2015-01-27

**Authors:** Giuliano Bonanomi, Guido Incerti, Gaspare Cesarano, Salvatore A. Gaglione, Virginia Lanzotti

**Affiliations:** Dipartimento di Agraria, University of Naples Federico II, via Università 100, Portici (NA), Italy; NERC Centre for Ecology & Hydrology, UNITED KINGDOM

## Abstract

Cigarette butts (CBs) are the most common type of litter on earth, with an estimated 4.5 trillion discarded annually. Apart from being unsightly, CBs pose a serious threat to living organisms and ecosystem health when discarded in the environment because they are toxic to microbes, insects, fish and mammals. In spite of the CB toxic hazard, no studies have addressed the effects of environmental conditions on CB decomposition rate. In this study we investigate the interactive effects of substrate fertility and N transfer dynamics on CB decomposition rate and carbon quality changes. We carried out an experiment using smoked CBs and wood sticks, used as a slow decomposing standard organic substrate, incubated in both laboratory and field conditions for two years. CB carbon quality changes during decomposition was assessed by ^13^C CPMAS NMR. Our experiment confirmed the low degradation rate of CBs which, on average, lost only 37.8% of their initial mass after two years of decomposition. Although a net N transfer occurred from soil to CBs, contrary to our hypothesis, mass loss in the medium-term (two years) was unaffected by N availability in the surrounding substrate. The opposite held for wood sticks, in agreement with the model that N-rich substrates promote the decomposition of other N-poor natural organic materials with a high C/N ratio. As regards CB chemical quality, after two years of decomposition ^13^C NMR spectroscopy highlighted very small changes in C quality that are likely to reflect a limited microbial attack.

## Introduction

Cigarette butts (CBs) are the most common type of litter on earth, with an estimated amount of 4.5 trillion discarded annually [1,2]. Unsurprisingly, several studies have reported that CBs are the most common item retrieved by clean-up activities in public areas such as beaches and parks [3,4]. Beyond being unsightly, when disposed of in the environment CBs pose a major threat to living organisms and ecosystem health (review in [5]). The few studies available report that CBs are toxic to microbes and cladocerans [6], insects [7], and also fish [8]. A recent study reported that CBs affect avian behaviour in urban ecosystems [9]. Such studies highlighted a higher toxic effect of smoked vs. unsmoked CBs since the former retain a substantial amount of nicotine and other compounds derived from tobacco combustion, including hydrogen cyanide, ammonia, acetaldehyde, formaldehyde, benzene, phenols and pyridines [10].

In addition to posing a toxic hazard, CBs accumulate in the environment in alarming quantities because of their slow degradation rate. They are made of compressed, plasticized cellulose acetate wrapped in an external paper layer. The high degree of acetate substitution (~2.45) makes the cellulose inaccessible to microbes for biological decomposition [11]. To become a food source for environmental microbes, cellulose acetate can be de-acetylated by chemical hydrolysis to a lower degree of substitution (~1), a fairly slow process under ambient conditions that is favoured by high UV radiation [12]. Despite the knowledge available about degradation of pure cellulose acetate films (review in [13]), few studies have addressed the degradation dynamics of whole CBs in realistic ecological conditions. According to grey literature studies, often sponsored by the tobacco industry, CBs require several years to degrade completely (e.g. [14,15]), but robust scientific data were not provided. In this regard, to the best of our knowledge, no peer-reviewed work has investigated long-term CB decomposition.

Another environmental factor that can slow down degradation of CBs is their low nutrient content, especially nitrogen (N). The regulatory role of N in litter decomposition has been extensively investigated for organic plant residues [16]. Decomposition is mainly controlled by temperature [17], water availability [18], and biochemical quality in terms of organic C types of N content [19]. In particular, N availability becomes ecologically important during decomposition when the C/N ratio of the decomposing substrate lies above a critical threshold of ~30–35. In this condition N starvation limits microbial activity [20] and decomposer microbes are able to get extra N from external sources (e.g. the underlying soil) and transfer it into the N-poor substrate to meet their nutritional requirements [21]. The very low initial N content of CBs (see the Results section) may well create intense microbial N starvation that can further limit microbial colonisation of both CB wrapping paper and the internal cellulose acetate filter. Under this assumption, exacerbated microbial N starvation should be expected in environments where nutrient availability is low (e.g. city pavements, railways, sandy beaches, etc). On the other hand, if N transfer from the surrounding environment (e.g. soil, water body sediment, etc.) significantly enhances the CB mass loss rate, this should be taken into account to better understand CB decomposition and predict CB residence time in different environmental conditions.

In the last decade, chemical throughput methods, including pyrolysis-gas chromatography/mass spectrometry [22], near-infrared reflectance spectroscopy [23] and ^13^C-cross-polarization magic angle spinning (CPMAS) nuclear magnetic resonance (NMR) spectroscopy [24], have been applied to characterise organic C-based materials at molecular level. ^13^C-CPMAS NMR has proved useful to provide a description of the total organic chemical C composition of complex matrices, such as plant litter [25], compost and peat [26], allowing the resonance signals of all the carbons of the analyzed samples to be obtained. Since the chemical shifts of different C atoms depend on their molecular environment, important information about their chemical type and the nature and number of substituents allows the attribution of observed carbons to a particular class of organic compounds. Here, by analysing CB samples at different decomposition stages, we assess the changes of different classes of organic C corresponding to different decay levels.

In this study, for the first time, we investigated the interactive effects of substrate fertility and N transfer dynamics on CB decomposition rate and carbon quality changes. We carried out an experiment with smoked CBs and wood sticks, used as a slow-decomposing standard organic substrate, incubated in both laboratory and field conditions. We tested the hypothesis that a net N transfer from N-rich soil to N-poor materials (i.e. CBs and wood sticks) enhances the substrate decomposition rate. In particular, an accelerated decomposition process is expected when CBs and wood sticks are decomposed in the presence of N-rich compared with N-poor soils. The specific aims of this work were to assess: (i) the dynamics of CB breakdown; (ii) the occurrence of N transfer from the surrounding environment to CBs, and (iii) whether and how CB carbon quality changes during decomposition by using ^13^C CPMAS NMR. Based on the above considerations, three main hypotheses were tested: (1) CBs show a low decomposition rate, which is slower than that of wood sticks; (2) a net N transfer occurs from N-rich soil to N-poor CBs and wood sticks; (3) a net N transfer promotes decomposition of both CBs and wood sticks.

## Materials and Methods

### Material collection

Regular filtered cigarettes from four common brands were purchased new and artificially smoked (for method details see [6]). As a comparative slow-decomposing standard organic substrate, we used wood sticks cut from *Q*. *ilex* branches (Portici 40° 48’ 43” N–14° 20’ 49” E, Southern Italy) as described in Bonanomi et al. [27]. The CBs and wood sticks showed different initial chemical features as follows (values are average ± standard deviations): i) cigarette butts (N content = 0.21 ± 0.12% of dry matter; C/N ratio = 192.39 ± 12.11; ii) wood sticks (N content = 0.10 ± 0.02%; C/N ratio = 440.75 ± 32.31; lignin content = 34.63 ± 4.54% of dry matter).

### Decomposition experiment

Organic matter decomposition in field conditions mainly depends on organic matter quality, water availability and temperature [16]. In our study, we decomposed organic substrates both in laboratory and field conditions. The laboratory experiment was included to reduce the importance of water availability and temperature on decay rate variation and to isolate the effect of CB biochemical quality.

Decomposition experiments were carried out according to the litterbag method [16]. CBs and wood sticks were cut with scissors to obtain pieces of 200 mg each. Litterbags were filled with 30 pieces of each material and then placed in microcosms in five different environmental conditions: i. laboratory without soil; ii. laboratory with grassland soil; iii. laboratory with sand dune soil; iv. grassland field, and; v. sand dune field. Grassland and sand dunes were selected because they are common natural environments where CBs are discarded, while showing large differences in soil texture and fertility. The main characteristics of sand dune soil were: sand 56.1%, silt 26.9%, clay 17.0% (sandy loam soil), pH 7.65, organic C 30.0 g/kg, total N 1.64 g/kg, C/N 18.3, total CaCO_3_ 224 g/kg, available phosphorus (P_2_O_5_) 28.3 mg/kg, exchangeable potassium 0.13 meq/100 g, exchangeable magnesium 5.11 meq/100 g, exchangeable calcium 18.6 meq/100 g, exchangeable sodium 0.40 meq/100 g, and EC 0.17 dS/m. For grassland the soil chemical characteristics were: sand 42.0%, silt 35.0%, clay 23.0% (loam soil), pH 8.01, organic C 16.19 g/kg, total N 3.90 g/kg, C/N 4.11, total CaCO_3_ 140 g/kg, available phosphorus (P_2_O_5_) 174.71 mg/kg, exchangeable potassium 0.62 meq/100 g, exchangeable magnesium 0.96 meq/100 g, exchangeable calcium 15.5 meq/100 g, exchangeable sodium 0.06 meq/100 g, EC 0.126 dS/m. No specific permits were required for the decomposition experiment at the two field study sites (i.e. Portici 40° 48’ 43” N–14° 20’ 49” E and Agropoli 40°25′10.11′′N 14°59′13.34′′, both located in the region of Campania, southern Italy). Moreover, the field studies did not involve endangered or protected species.

In the laboratory, litterbags were placed in a growth chamber under controlled temperature (22 ± 2°C night and 25 ± 2°C day) and water (watered with distilled water every seven days to water holding capacity, previously determined equal to 260%) conditions. The full experimental design entailed five treatments (i.e. environmental conditions for incubation) for CB and wood sticks, replicated ten times for each of eight planned dates of retrieval from the start of the experiment (i.e. 30, 90, 180, 360, 720, 1080, 1800, and 3600 days), for a total of either 12000 butts or wood sticks. At the moment the experiment is still running and herein we present the results for substrates retrieved after 30, 90, 180, 360, and 720 days of decomposition. After harvesting, the substrates collected were oven-dried (40°C until constant weight was reached) and weighed afterwards to the nearest 0.001 g.

### Chemical analyses

Smoked CBs, both undecomposed and decomposed for 720 days were characterized for total C and N content by flash combustion of microsamples (5 mg each) in an Elemental Analyser NA 1500 (Carlo Erba Strumentazione, Milan, Italy). Moreover, undecomposed CBs as well as CBs decomposed for 720 days were characterised by ^13^C-CPMAS NMR [24] obtained in solid state and under the same conditions, thus allowing comparative analysis of the resulting spectra. In addition, to highlight the differences between cigarette butts, mainly made of cellulose acetate, and pure cellulose, filter paper cellulose also underwent ^13^C-CPMAS NMR analysis. The spectrometer used was a Bruker AV-300 equipped with a 4 mm wide-bore MAS probe (for further details see [28]). Spectral regions and corresponding C types were identified following Pane et al. [26]: 0–45 ppm = alkyl C; 46–60 ppm = methoxyl and N-alkyl C; 61–90 ppm = O-alkyl C; 91–110 ppm = di-O-alkyl C; 111–140 ppm = H- and C- substituted aromatic C; 141–160 ppm O-substituted aromatic C (phenolic and O-aryl C); 161–190 ppm carboxyl C. Concerning the 161–190 ppm region, the carboxylic C term was used to indicate the absorption of carboxylic acids and their ester and amide derivatives. Finally, the degree of acetylation of cellulose assessed by dividing the integral of the metyl C signal by the C-1 signal.

### Data analysis

We used general linear models (GLMs) to test main and second order interactive effects of litter type (either CBs or wood sticks), type of soil addition (either grassland or sand dune soil), temperature and water conditions (either controlled in the laboratory or natural conditions in the field), and decomposition time (treated as a continuous covariate) on litter mass loss. In order to provide a detailed assessment of environment-related interactions, we expressed the dependent variable as the difference of percent mass loss compared to the control. For a given material decomposing for a given number of days, we considered as the control the same material, incubated for the same number of days in controlled conditions without soil addition.

CB nitrogen content after 720 days of decomposition and variation of organic C types corresponding to NMR spectral regions were assessed by one-way ANOVA. Differences at each level of treatment were statistically evaluated by post-hoc Duncan’s test. Significance was evaluated in all cases at *P* < 0.05 and < 0.01. Nitrogen transfer was indirectly assessed by total N mass balance after 720 days of decomposition, following [27]. Total N content was determined in undecomposed CBs and wood sticks (i.e. initial values) and after 720 days of incubation, and, for each material, expressed as percentage of the corresponding initial value. During decomposition, a value significantly higher than 100 indicated an increase of total N content, and was used as indirect evidence of N transfer from the soil to the decomposing material. Significant deviations from 100 were assessed by t-test for single means. The level of statistical significance was corrected for multiple comparison by applying the Bonferroni’s correction. The level of statistical significance was set to *P* = 0.05/N, with N = 10 being the total number of performed tests.

## Results

### Cigarette butt decomposition and N dynamics

Mass loss of the tested materials was significantly affected by incubation conditions (S1 Table). Besides litter type (i.e. CBs and wood sticks) and incubation time, a direct significant effect was also recorded for the type of soil addition, but not for the regime of temperature and water conditions (S1 Table). However, observations related to the incubation conditions were not limited to simple and direct effects, with GLM analysis showing significant interaction terms for almost all tested parameters.

**Figure 1:**
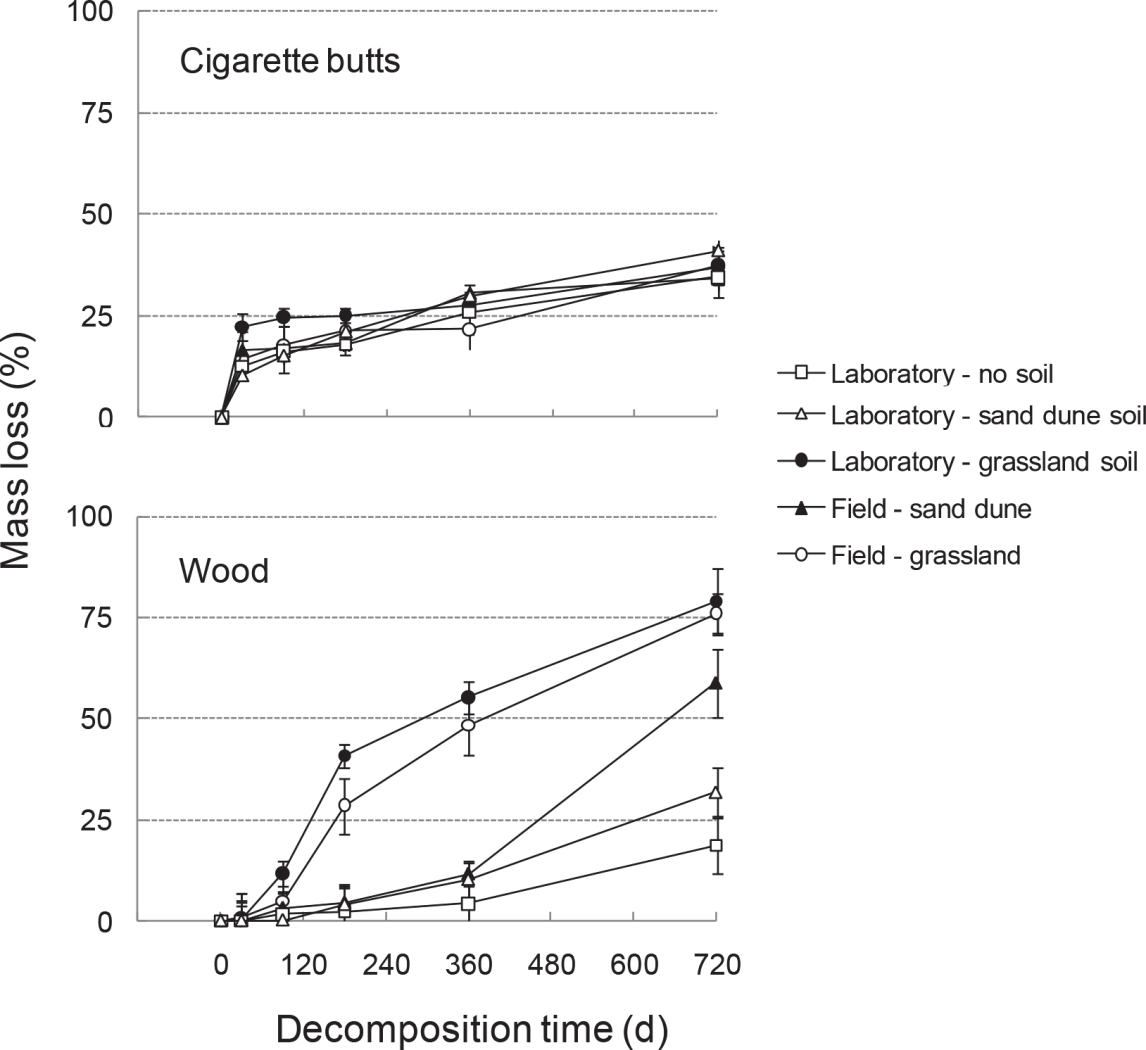
CB and wood sticks mass loss. Mass loss during a 720-day decomposition period for cigarette butts (above) and wood sticks (below) in laboratory and field conditions. Data refer to mean ± standard deviation of 25 replicates for each material (for statistics see S1 Table).

**Figure 2:**
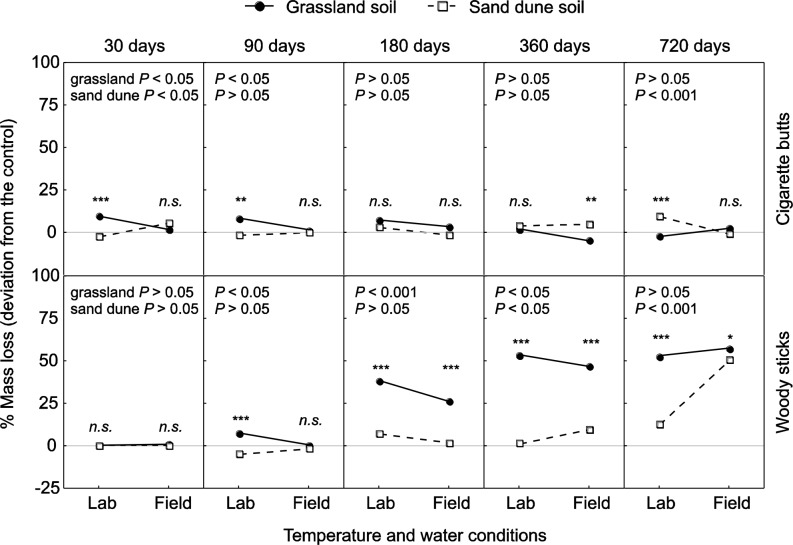
Interactive effects of environment conditions on mass loss. Interactive effects of environment-related parameters on percent mass loss of the tested materials (CBs, top panel row, and wood sticks, bottom panel row) at different decomposition stages (from 30 to 720 days, panel columns). On each graph, *P*-values in the top-left corner refer to the effects of temperature and water treatment (controlled in the laboratory, natural conditions in the field) on samples incubated with either grassland or sand dune soil. Stars above symbols refer to the effects of soil type within each temperature and water treatment (***: *P* < 0.001, **: *P* < 0.01, *: *P* < 0.05, n.s.: *P* > 0.05). All statistical results refer to post-hoc Duncan tests from GLM in S1 Table.

For CB, a rapid mass loss (~15–20% of initial mass) was observed in the first thirty days of incubation, being slightly faster in laboratory conditions over grassland soil (Figs. 1 and S1). However, as decomposition proceeded the rate of mass loss dramatically declined, with a total mass loss of only ~30–35% after 720 days (Fig. 1). Differences among the experimental treatments, compared to the control incubated in controlled conditions and without soil addition, were highly significant only at the early and very late stages of the decomposition process, corresponding to 30 and 720 days, respectively. In particular, after 30 days of decomposition CBs incubated in the field with grassland soil showed a significantly higher mass loss compared to samples incubated in controlled conditions (Fig. 2), while the opposite trend was observed for CBs treated with sand dune soils (Fig. 2). Variations of percent mass loss compared to the control were also significantly different between the samples incubated with the two types of soil in the laboratory, but not between those decomposing in the field (Fig. 2). After two years of decomposition a different pattern was found, with CBs incubated with sand dune soil showing highest mass loss in the laboratory compared to field conditions, while samples decomposing with grassland soil were unaffected by temperature and water conditions (Fig. 2). At the intermediate incubation stages (90 to 360 days) most of the tested effects were either not significant, or weaker than those described above. Remarkably, deviation of mass loss compared to the control was always lower than 10% in all CB samples, irrespective of the incubation conditions and the related significant effects (Fig. 2).

In the case of the wood sticks decomposition was initially slower compared to CBs (Fig. 1). However, large differences in mass loss of wood sticks emerged with time among experimental treatments. Mass loss was very low in the laboratory without soil and with sand dune soil. In contrast, a high mass loss (over 75% of the initial mass) was recorded in the laboratory with grassland soil and in grassland field (Fig. 1). Intermediate mass loss was observed for wood sticks incubated in a sand dune field (Fig. 1). Differences among the experimental treatments, compared to the control incubated in controlled conditions and without soil addition, dramatically increased with decomposition time, depicting a completely different pattern with respect to CBs (Fig. 2). In particular, at the early decomposition stage the mass loss of wood sticks did not differ from the control, irrespective of the experimental conditions. Then, as decomposition proceeded, mass loss progressively increased, with peculiar different patterns related to the conditions of incubation (Fig. 2). In general, samples incubated with grassland soil showed significantly higher mass loss when incubated in controlled conditions compared to decomposition in the field, with the effect almost disappearing after 720 days (Fig. 2). Moreover, addition of grassland soil produced higher mass loss compared to sand dune soil, with only one exception (i.e. after 90 days of incubation in the field, Fig. 2). Finally, addition of sand dune soil did not produce significant effects on mass loss of wood sticks during the first year of the experiment, while after 720 days samples incubated in the field showed significantly higher mass loss compared to those decomposing in controlled conditions (Fig. 2).

**Figure 3:**
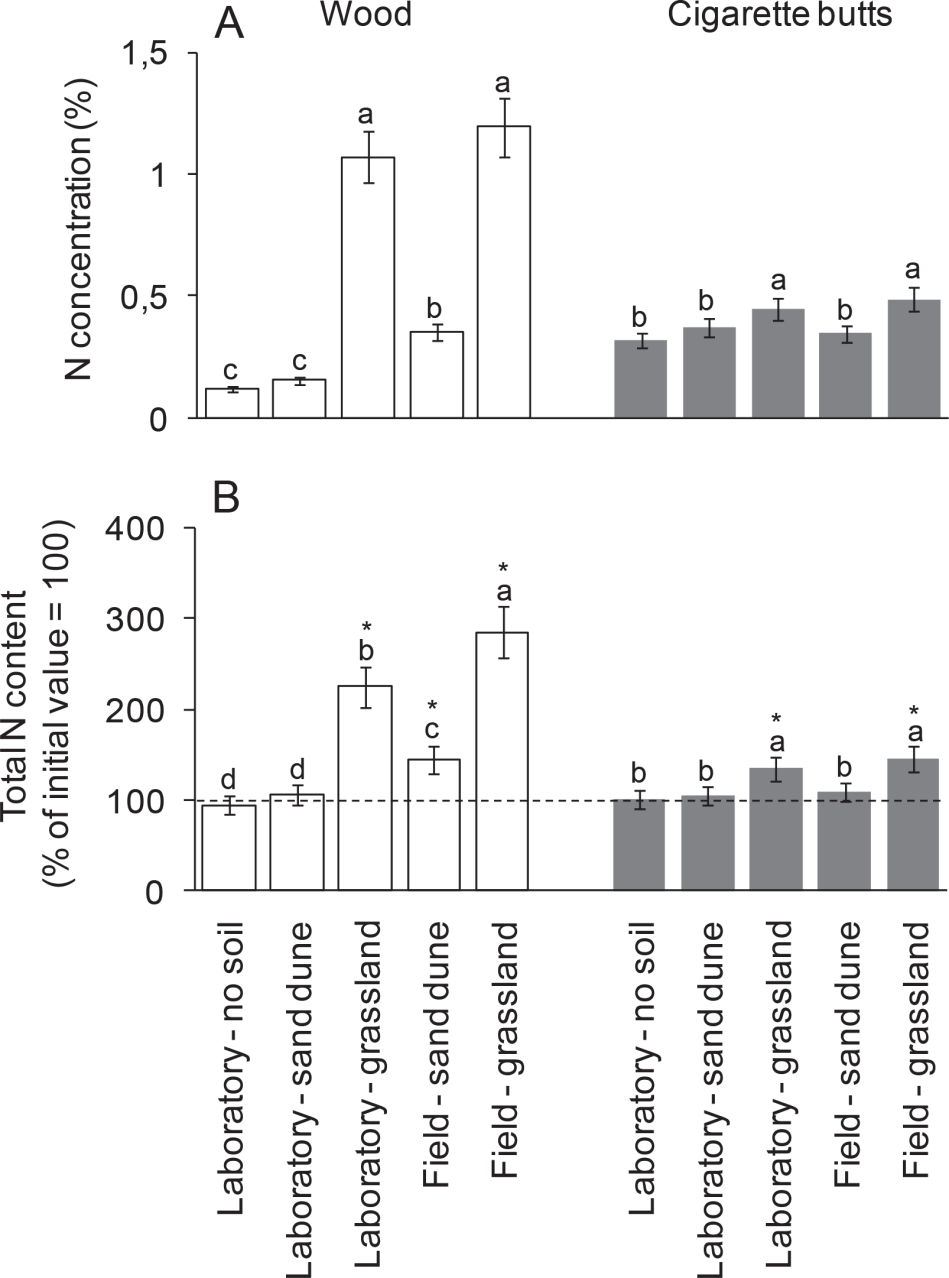
Nitrogen concentration and transfer in wood sticks and cigarette butts. Nitrogen concentration (A, expressed as percentage) and total content (B, expressed as a percentage of initial value fixed at 100%) after 720 days of decomposition in laboratory and field conditions in wood sticks and cigarette butts. Initial N concentration was 0.10% and 0.21% for wood sticks and cigarette butts, respectively. Data refer to mean ± standard deviation; different letters indicate statistically significant differences within each material (Duncan’s test at *P* < 0.05). Materials showing total N content significantly higher than 100 (t-test with Bonferroni’s correction for multiple comparison, *P* < 0.005) are marked with * indicating N transfer from the soil.

After 720 days of incubation, N concentration increased in both wood sticks and CBs (Fig. 3). In detail, in both materials N concentration was highest when incubated with grassland soil in field conditions, and slightly lower in the laboratory (Fig. 3). In the case of wood sticks decomposing in presence of sand dune soil, N concentration was higher under field conditions compared to laboratory conditions (Fig. 3). For both materials, incubation in absence of soil produced the lowest N concentration, although not significantly different from that observed in presence of sand dune soil in controlled conditions (Fig. 3).

Total N content, assessed by C and N mass balance, after 720 days of decomposition was roughly constant for CBs incubated without soil and with sand dune soil, while a slight but significant increase was observed for CBs incubated with grassland soil both in laboratory and field conditions (Fig. 3). Total N content increased in wood sticks incubated with grassland soil and, albeit to a lesser extent, in sand dune field (Fig. 3).

### Cigarette butt chemical changes

**Figure 4:**
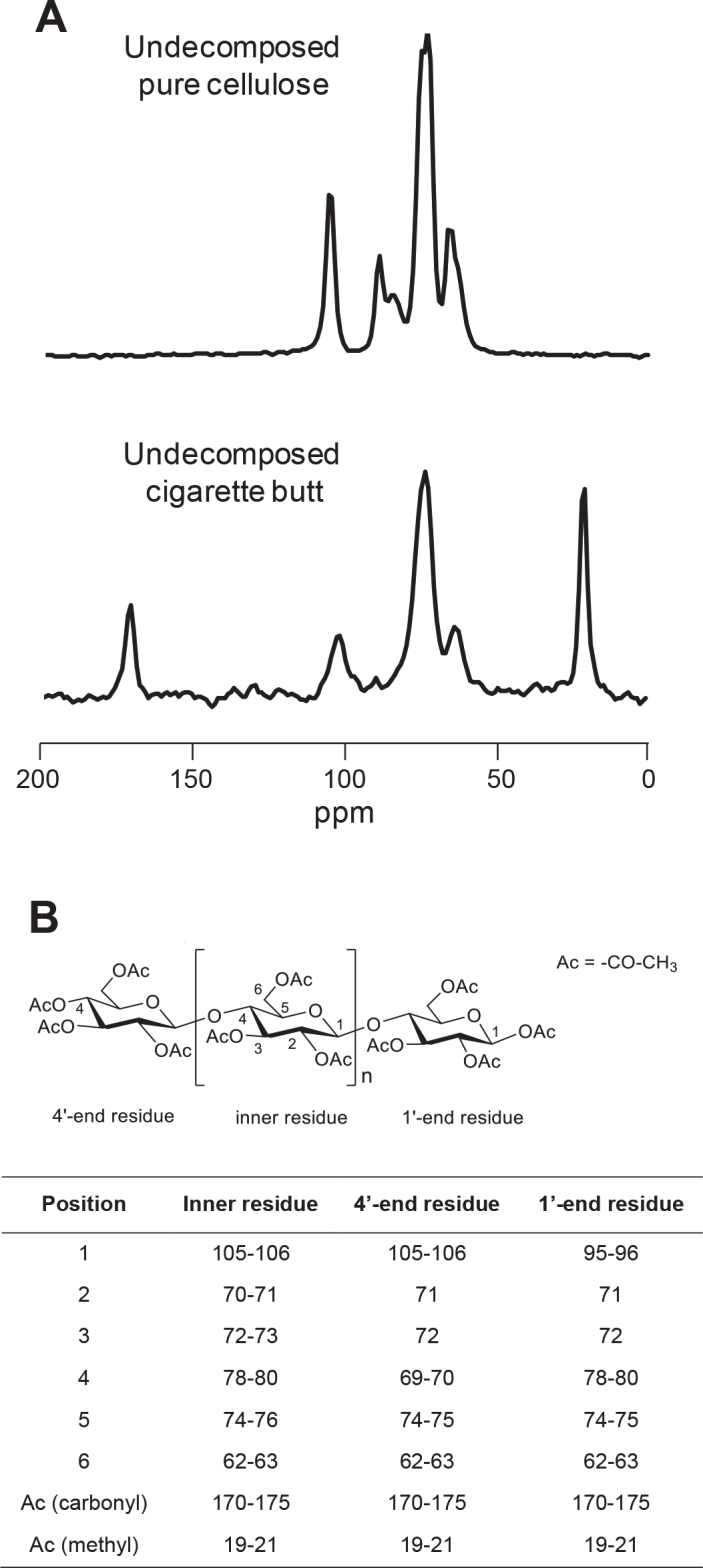
CB biochemistry assessed by ^13^C CPMAS NMR. ^13^C CPMAS NMR spectra of undecomposed pure cellulose paper filter and smoked cigarette butts (A). ^13^C CPMAS NMR data of acetyl-cellulose are also reported (B). The assignments were obtained by comparing our data with those reported in the literature for glucose acetate [29].

**Figure 5:**
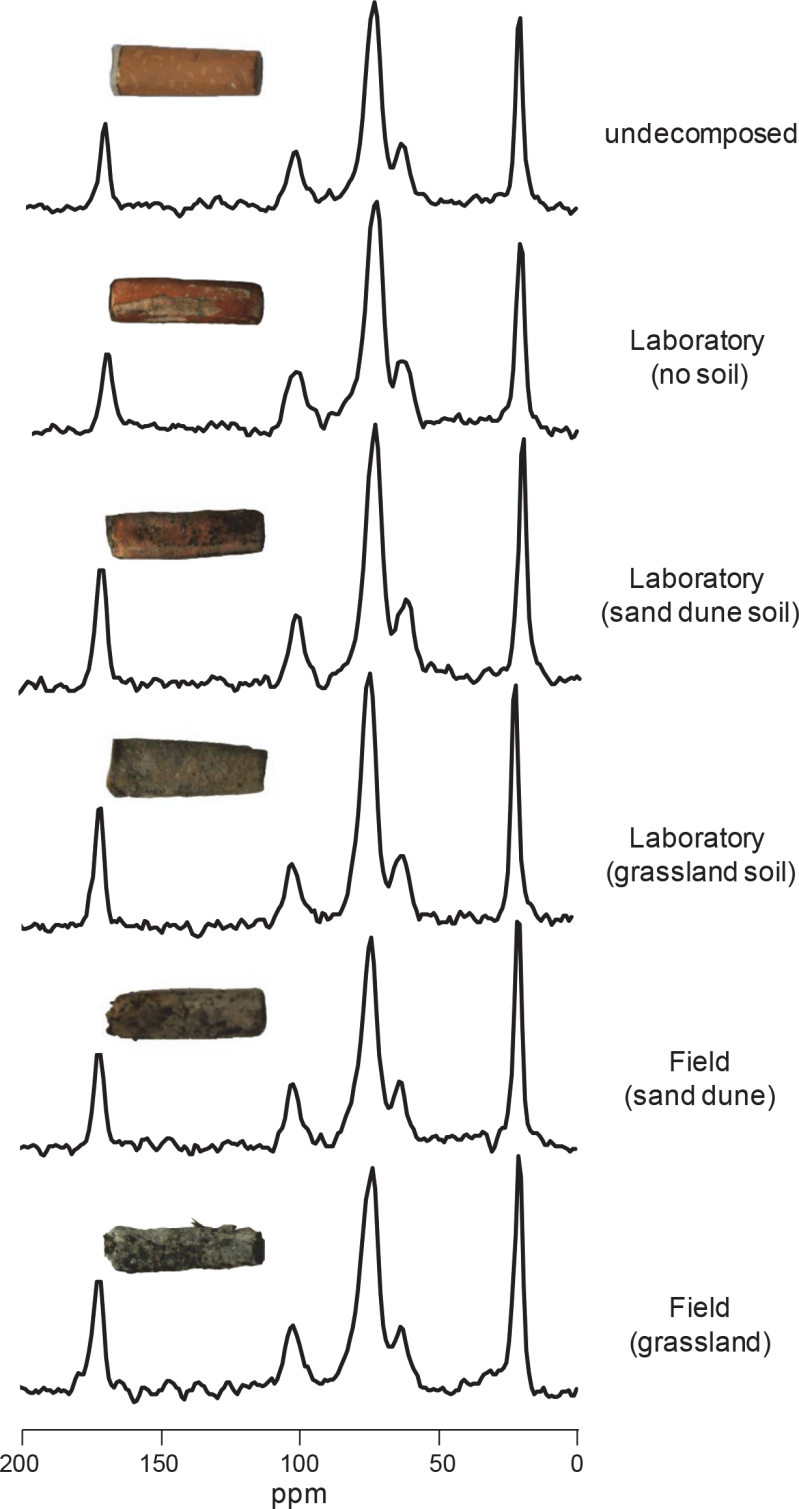
Variation in carbon biochemical quality assessed by ^13^C CPMAS NMR. Spectra refer to cigarette butts either undecomposed or after 720 days of decomposition in laboratory and field conditions. Insets in each spectra shows pictures of a cigarette butt at the corresponding decomposition stage.

**Figure 6:**
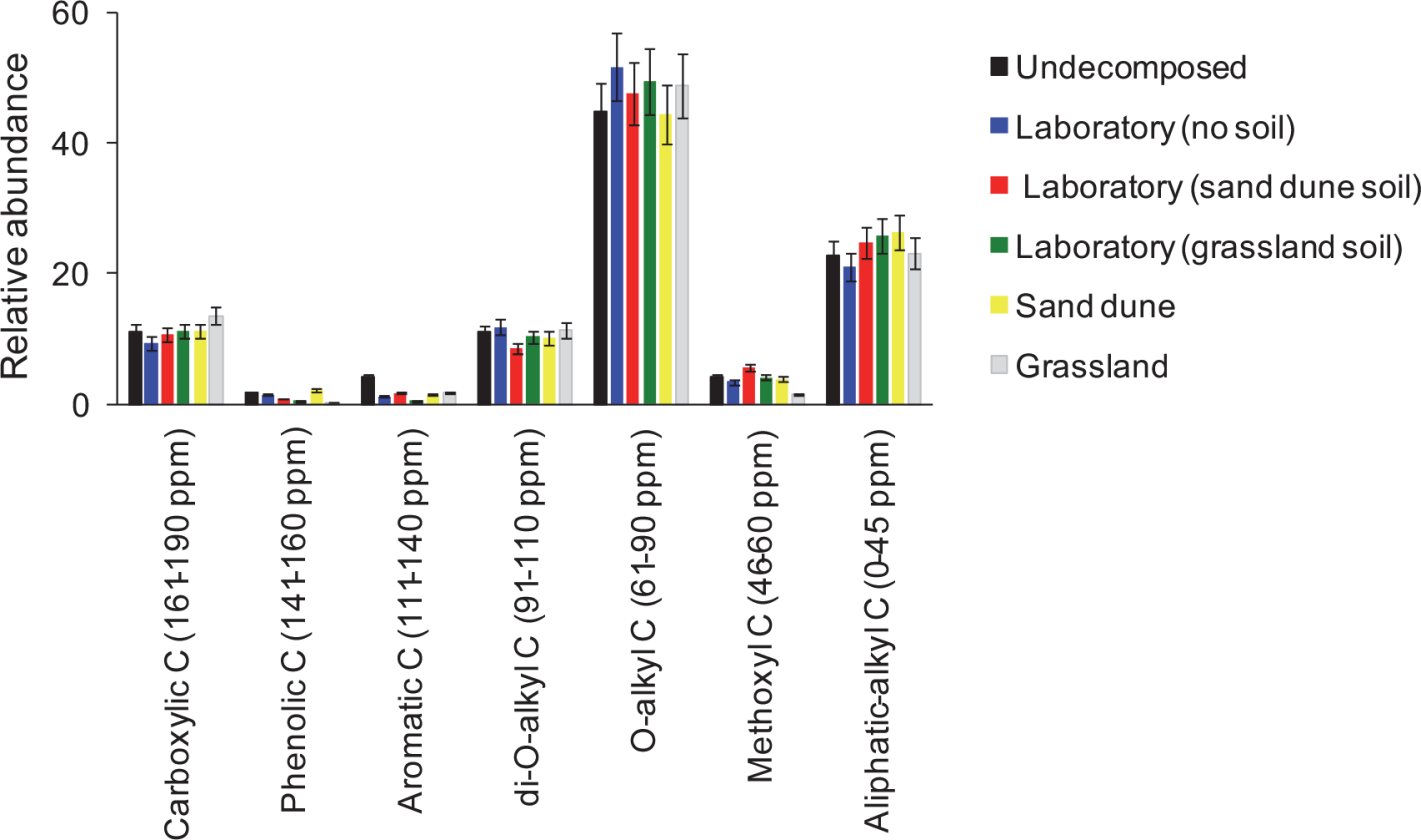
Assessment of CB biochemical quality variation during decomposition. Variation in seven main classes of organic carbon assessed by ^13^C-CPMAS NMR spectroscopy in undecomposed cigarette butts and after 720 days of decomposition under laboratory and field conditions. Data refer to mean ± standard deviation (*N* = 3). No statistically significant differences were recorded (Duncan’s test at *P* < 0.05).

Undecomposed, smoked CBs showed considerable differences with pure cellulose paper as highlighted by ^13^C NMR spectra (Fig. 4): CBs showed peaks of glucose residues at δ 62–63 (C-6), 69–76 (C-2/C-3/C-4/C-5) and 105–106 (C-1) (Fig. 4). In addition, two major peaks at δ 19–21 and 170–175, present only in the CB spectra, were attributed to methyl and carboxyl carbon signals, respectively, of acetyl groups and thus reflected the degree of acetylation of cellulose. The degree of acetylation was 2.38 for undecomposed CBs and, in average, 2.32 for CBs decomposed in different environmental conditions. After 720 days of decomposition, CBs showed small chemical changes (Figs. 5, 6): no changes in *O*-alkyl C, mainly associated to sugars and polysaccharides, nor in alkyl C, related to acetyl groups were observed. By contrast, a slight decrease in aromatic and phenol C regions (111–140 ppm and 141–160 ppm, respectively) were recorded. This signal variation is attributable mainly to other classes of minor compounds of phenolic and aromatic origin, present in CBs that are lost during decomposition. In this regard, nicotine residues may give a contribution in this ^13^C NMR region. Concerning the signals of cellulose acetate, no significant variation was observed for its carbon signals in ^13^C NMR spectra. In particular, degradation of ester bonds would result in the loss of acetate from cellulose acetate and thus should give a loss of intensity of the signals at δ 19–21 and 170–175 (Fig. 4). This was not observed in the spectra of decomposed material (Figs. 5, 6), indicating that this bond is not subject to degradation in the first two years of incubation. Furthermore, the break at the level of interglycosidic linkage was also excluded by the analysis of the spectra because the signals of acetalic anomeric carbons at δ 105–106 showed no change in the ^13^C NMR spectra of decomposed material, indicating that the anomeric carbon is involved in glycosidic bond and is not free in the hemiacetalic form [29].

## Discussion

Our experiment confirmed the low degradation rate of CBs which, on average, lost only 37.8% of their initial mass after 2 years of decomposition. A net N transfer occurred from soils to CBs but, contrary to our hypothesis, mass loss in the medium-term (two years) was not affected by N availability in the surrounding substrate. The opposite held for wood sticks, in agreement with the model that the presence of an N-rich substrate promotes the decomposition of other, N-poor natural organic materials with a high C/N ratio [27,30]. Finally, by using ^13^C CPMAS NMR we observed minimal CB carbon quality changes during decomposition.

### Cigarette butt decomposition

A rapid initial CB mass loss, on average accounting for 15.2% of starting mass, was observed in the first thirty days of decomposition. This pattern, after 30 days of incubation, was completely different from that of wood sticks, both in terms of magnitude (mass loss of wood sticks was only 0.3% in the same decomposition phase) and variability, with mass loss of CBs, but not of wood sticks, being significantly affected by both soil type and temperature and water conditions. This initial rapid mass loss may be ascribed to leaching of soluble materials and to the decomposition of the external paper layer wrapping the internal filter. This hypothesis is corroborated by the observed rapid disappearance of the wrapping paper layers (S1 Fig.) and by the observation that the wrapping paper represent in our samples 25.72% of total CB mass. In addition, a previous report showed no initial rapid mass loss for CBs devoid of the wrapping paper layer [14]. Moreover, early decomposition of CBs, but not of wood sticks, was diversely affected by the different environmental conditions mimicked by our experimental treatments. This evidence indicates early decaying of labile external paper more responsive to external temperature and water availability variations, compared to the recalcitrant, slow-decomposing wood sticks. Analogously, a higher sensitivity of fast-decomposing litter to shifts in temperature and water availability, compared to slow-decomposing litter, was found by [31] in the case of plant residues decomposing under Mediterranean climatic conditions.

In this regards, specific attention should be paid to the role of UV-light in CBs degradation. Recent studies demonstrated that UV can play an important stimulative role in degradation of plant litter, especially of lignin rich plant debris [32] in arid and semi-arid ecosystems (e.g. [33]). Our study was not specifically designed to directly address this issue, because we tested two sets of environmental conditions (i.e. natural and controlled conditions) manipulating water availability and temperature, but not directly controlling UV regimes. However, UV intensity and daily exposure were certainly lower in the laboratory than in open grasslands where UV directly reached the soil surface where CBs were placed. Then, our observation of CBs mass loss not significantly different between open grassland and grassland soil incubated in the laboratory, is consistent with two different hypotheses: first, none of the environmental parameters significantly affected CBs decay. Second, the effects of UV, temperature and water were mutually counterbalanced. In the latter case, in open field, under more limiting water and temperature conditions, the UV regime would have enhanced CBs decomposition, while in the laboratory the optimal controlled conditions of water availability and temperature would have been coupled with a reduced effect of UV.

The initial rapid CB mass loss was followed by a very slow decay rate observed thereafter: only an additional 11.9% and 9.7% of initial mass was lost in the subsequent incubation phases, lasting from 30 to 360 days and from 360 to 720 days of incubation, respectively. This decomposition pattern mirrored that observed in some plant leaf litter where the labile C fraction is rapidly decomposed, leaving preserved the recalcitrant fractions (e.g. lignin) requiring much longer for disappearance [16]. The very low CB mass loss can be related to resistance to microbial attack of its main component, i.e. cellulose acetate with a substitution degree of ~2.45. As for undecomposed CBs, the integration of methyl signals of acetyl groups over glucose C-1 signals gave a calculated substitution degree of 2.32, indicating that cellulose was still not significantly de-acetylated. To become a food source for environmental microbes, cellulose acetate can be de-acetylated by hydrolysis to a lower degree of substitution (~1), a rather slow physico-chemical process under ecologically relevant environmental conditions [12].

A further hypothesised explanation for the low CB decomposition rate concerns an N shortage that limits microbial activity. In general terms, microbial communities during litter decomposition can actively scavenge nutrients, especially N, from the surrounding environment to meet their requirements when the decomposing litter has a high C/N ratio, usually above 25–30 [21]. This process results in N immobilization within the microbial biomass [34]. In our case all substrate types (CBs and wood sticks) had a C/N ratio well above the mentioned threshold. Thus a combination of low chemical quality for microbial attack (i.e. cellulose acetate for CBs and high lignin content for wood) and environmental constraints in terms of N limitation may be the main causes of the slow decay rate observed. In other words, the very low initial N content of CBs and wood sticks is likely to create extreme microbial N starvation that could be relaxed once external N is provided. In this regard, accelerated decomposition of low quality litter in plant residue mixtures has been attributed to a net transfer of nutrients, especially N, from a source substrate to a target, usually nutrient-poor material [27,35]. Our study provides direct support for such a model only for wood sticks. Indeed, we found an increase in N concentration as well as total N content for both CBs and wood sticks when they were paired with N-rich grassland soil. Although increase in N concentration can be directly related to N preservation and organic C degradation, the increase of total N content is consistent with a net transfer of N from the surrounding substrate to wood sticks and, to a lesser extent, to CBs.

N transfer among decomposing plant litters in mixture has been previously reported and related to different processes including passive diffusion, through leaching and diffusion, or active transfer by fungal mycelia networking [36]. Such evidence suggests the occurrence of microbial N starvation that presumably limits microbial decomposition. However, N transfer from soil to the decomposing substrate is translated into an increased mass loss for wood sticks but not for CBs. The different mass loss response of CB and wood to N transfer can be ascribed to the C quality of the substrates and, with more detail, to the high lignin content of wood and the plasticized cellulose acetate of CBs. At the early decay phases, high N availability may sustain microbial activity to consume labile C compounds rapidly, resulting in a rapid mass loss rate. In our study, N transfer could explain the faster mass loss of CBs observed at the early decomposition stage, probably relieving N starvation and enhancing decomposition of the cellulose wrapping layer. Thereafter, the decomposition of CBs was not responsive to N content changes. This result suggests that CB decomposition sensitivity to N availability can be dramatically reduced in the presence of biochemical constraints to microbial attack, such as the high degree of substitution of cellulose acetate. However, it may be hypothesised that as cellulose acetate is progressively chemically de-acetylated, CBs would become progressively sensitive to nutrient availability in the surrounding substrate. Future studies, including data from our ongoing long-term experiment, may provide useful insights to assess the impact of N transfer from soil to CBs upon cellulose acetate decomposition at later stages.

### Cigarette butt chemical changes

As regards CB chemical quality, here, for the first time, we describe biochemical changes of C types occurring after two years of decomposition using ^13^C NMR spectroscopy. The most interesting results are the very small spectral changes shown by CBs when decomposed under different environmental conditions. This contrasts with the significant changes observed during decomposition of plant-derived materials (review in [24]). Indeed, several studies concerning plant litter reported a considerable reduction in carbohydrates (*O*-alkyl C) and a concomitant increase in alkyl C, indicative of a progressive increase in organic matter bio-stability [19,24,25,37]. The almost complete absence of changes in C quality for CBs could be explained by a limited microbial attack that was unable to consume the carbohydrate fraction or to produce, as a result of microbial degradation, by-products and spoilage that would otherwise lead to a progressive accumulation of aliphatic compounds. However, a slight decrease was observed in the resonance regions at 111–140 ppm and 141–160 ppm. These signals variations can be attributable to classes of compounds bearing phenolic and aromatic carbons. Among these, chemical residues probably derived from tobacco combustion (e.g. nicotine, ethyl phenol) might play a major role. Interestingly, a fraction of these compounds present in undecomposed CBs is partially lost during decomposition, indicating a partial retention by the filter of CBs. Although the role of compounds derived from tobacco combustion in CBs eco-toxicology is well established [5], their role on CBs decomposition is unknown. Future studies can address this issue by comparing in decomposition experiments smoked *vs* unsmoked CBs. A previous study using ^13^C NMR spectroscopy demonstrated that the fungal-derived enzyme acetyl esterase specifically cleaved off the acetyl substituents from the C2- (at 168 ppm) and C3-position (at 169 ppm) from cellulose acetate with a degree of substitution < 1.8 [38]. In our study, however, no significant changes were observed between undecomposed and two-year-old CBs in the area ranging from 161 to 190 ppm corresponding to the carboxyl C region. This result suggests that two years of decomposition are not sufficient for significant de-acetylation of compressed, cellulose acetate with a degree of substitution of ~2.45. Future ^13^C NMR analyses of CBs decomposed for 3, 5 and 10 years from our ongoing experiment will be useful to ascertain how and when CBs become de-acetlyate, and if after this process this relatively simple substrate become a suitable food source for soil microbes. Our future data will be useful also to assess the chemical changes that CBs will undergo during long-term decomposition.

## Conclusions

Our experiment demonstrated very slow CB decomposition which, after the early decay of the small labile fraction, corresponding to the external paper layer, proceeds very slowly irrespectively of the environmental conditions occurring during the process. A net N transfer occurred from soils to CBs but, contrary to our expectations, mass loss after two years was unaffected by N availability in the surrounding soil. These results indicate that the chemical composition of CB cellulose is the most important factor affecting its mass loss, a factor that largely exceeds the impact of environmental conditions. As regards the chemical quality of CBs, after two years of decomposition ^13^C NMR spectroscopy highlighted very small changes in C quality. This probably reflects a limited microbial attack during the first two years of decomposition. Analyses of CBs from our ongoing experiment will be useful to reveal whether, when and to what extent CBs can become a C source for microbes and how they transform this material during decomposition. Future research can be directed to address the role of other factors possibly affecting CBs decomposition and not considered in the present study. In particular, alteration of the CBs surface by human smokers could be considered, including occurrence of organic debris (i.e. unburnt tobacco residues, compounds derived from tobacco combustion, gloss etc) as well as the specific microbial community deriving from human oral cavity [39] and their interactions with soil microbial communities. In the case of oral cavity microbes, a negligible role may be hypothesized, based on their relatively low taxonomical and functional diversity [40,41] compared to soil microbial communities [42,43]. On the other hand, specific attentions should be paid to the role of UV-light in CBs degradation, considering that recent finding reports an important role of this ecological factor in degradation of litter in arid and semi-arid ecosystems [33], especially of lignin rich plant debris [32].

## Supporting Information

S1 Fig CB images during decomposition. Selected images of cigarette butts after 30, 180, and 720 days of decomposition incubated in different environmental conditions.
(TIF)

S1 Table Summary of the GLM of the decomposition experiment. Summary of the GLM testing for main and 2nd order interactive effects of litter type, temperature and water conditions, type of soil addition and decomposition time on percent mass loss, expressed for each material as deviation from the control (i.e. the same litter type, incubated for the same number of days in controlled conditions without soil addition). Asterisks indicate statistical significance (***: *P* < 0.001; **: *P* < 0.01; *: *P* < 0.05; n.s.: *P* > 0.05).
(DOC)
