# Supplementary figures and images for: Cigarette Butt Decomposition and Associated Chemical Changes Assessed by 13C CPMAS NMR
Source: PLoS One. 2015 Jan 27;10(1):e0117393. doi: 10.1371/journal.pone.0117393 (PMC4307979; doi:10.1371/journal.pone.0117393)

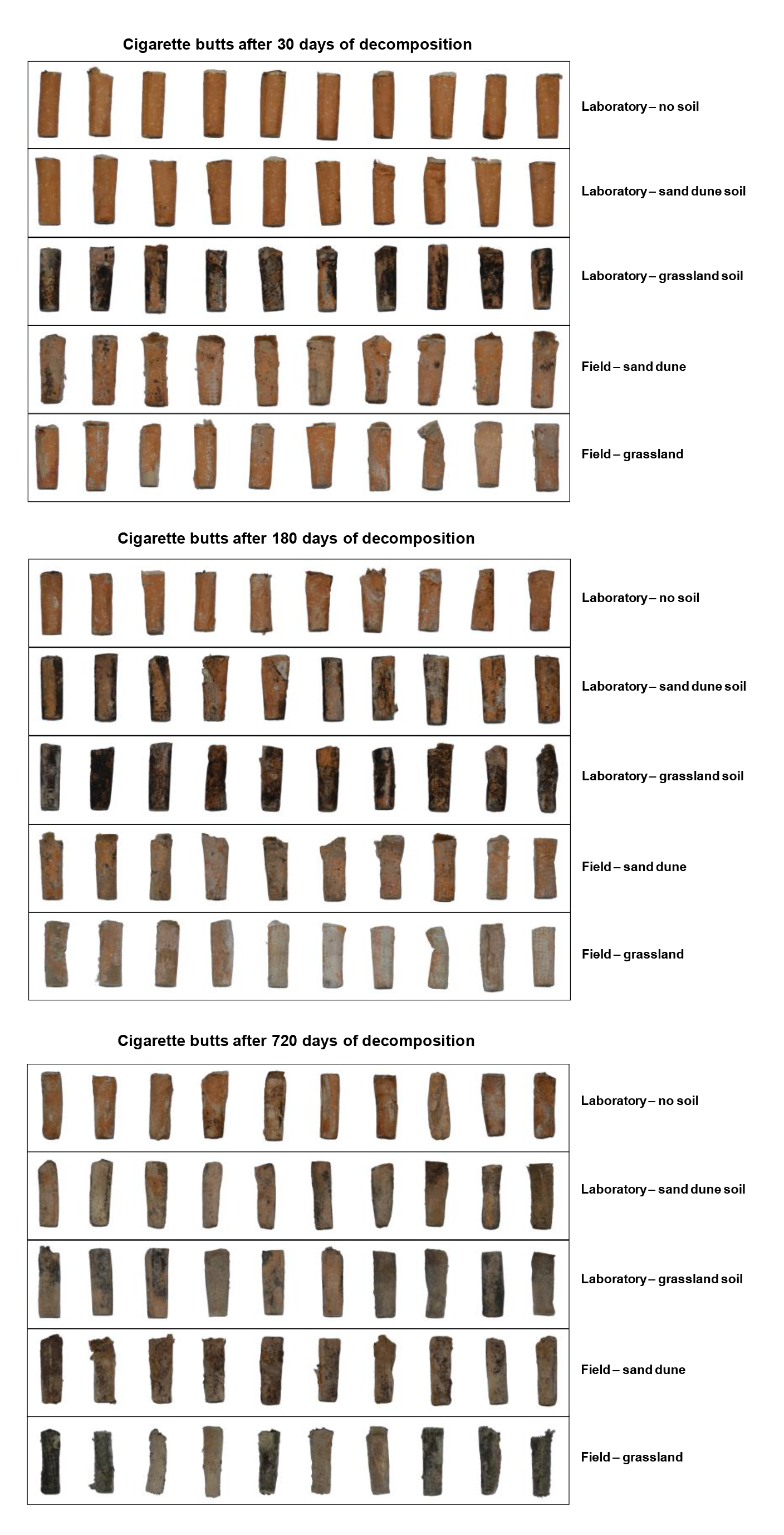

Supplement: S1 Fig — Selected images of cigarette butts after 30, 180, and 720 days of decomposition incubated in different environmental conditions. (TIF) [file pone.0117393.s001.tif]
