# Supplementary material for: Cigarette Butt Decomposition and Associated Chemical Changes Assessed by 13C CPMAS NMR
Source: PLoS One. 2015 Jan 27;10(1):e0117393. doi: 10.1371/journal.pone.0117393 (PMC4307979; doi:10.1371/journal.pone.0117393)
Supplement: S1 Table — Summary of the GLM testing for main and 2nd order interactive effects of litter type, temperature and water conditions, type of soil addition and decomposition time on percent mass loss, expressed for each material as deviation from the control (i.e. the same litter type, incubated for the same number of days in controlled conditions without soil addition). Asterisks indicate statistical significance (***: P < 0.001; **: P < 0.01; *: P < 0.05; n.s.: P > 0.05). (DOC) [file pone.0117393.s002.doc]

|  | d.f. | SS | MS | F | *P* | Statistical  significance |
| --- | --- | --- | --- | --- | --- | --- |
| Litter type (L) | 1 | 257.61 | 257.61 | 5.917 | 0.015417 | * |
| Temperature and Water conditions (TW) | 1 | 24.60 | 24.60 | 0.565 | 0.452642 | n.s. |
| Soil type (S) | 1 | 5197.97 | 5197.97 | 119.385 | < 0.000001 | *** |
| Decomposition time (Time) | 1 | 14528.84 | 14528.84 | 333.692 | < 0.000001 | *** |
| L x TW | 1 | 260.81 | 260.81 | 5.990 | 0.014798 | * |
| L x S | 1 | 4737.65 | 4737.65 | 108.812 | < 0.000001 | *** |
| TW x S | 1 | 473.49 | 473.49 | 10.875 | 0.001058 | ** |
| L x Time | 1 | 16507.18 | 16507.18 | 379.130 | < 0.000001 | *** |
| TW x Time | 1 | 31.49 | 31.49 | 0.723 | 0.395591 | n.s. |
| S x Time | 1 | 476.32 | 476.32 | 10.940 | 0.001023 | ** |
